# Effective analysis of testicular seminoma toxicity and mechanisms of acetyl tributyl citrate using network toxicology, bulk RNA sequencing data, single-cell RNA sequencing data, and clinical data

**DOI:** 10.3389/fimmu.2026.1752528

**Published:** 2026-07-13

**Authors:** Haisheng Yi, Yibo Gong, Wenjie Yang, Yinzhao Jia, Jintao Chen, Chao Zhang, Xueming Lin, Hua Tian, Bo Wu, Xiaoming Cao, Gang Liang, Xiaobin Yuan

**Affiliations:** 1 Department of Urology, First Hospital of Shanxi Medical University, Taiyuan, Shanxi, China; 2 First College of Clinical Medicine, Shanxi Medical University, Taiyuan, Shanxi, China; 3 Department of Operating Room, First Hospital of Shanxi Medical University, Taiyuan, Shanxi, China; 4 Department of Pathology, First Hospital of Shanxi Medical University, Taiyuan, Shanxi, China

**Keywords:** acetyl tributyl citrate, prognostic genes, risk model, single-cell RNA sequencing, testicular seminoma

## Abstract

**Introduction:**

Acetyl tributyl citrate (ATBC) is a widely used plasticizer ubiquitously present in the environment. Long-term high-dose ATBC exposure has been associated with reproductive system damage, yet its toxic effects and underlying mechanisms in testicular seminoma remain unclear. This study aimed to investigate the impact of ATBC on the prognosis of testicular seminoma.

**Methods:**

We integrated network toxicology analysis, single-cell RNA sequencing (scRNA-seq), bulk RNA-seq data, and clinical datasets to explore the prognostic role of ATBC-related genes in testicular seminoma. Bulk RNA-seq profiles, scRNA-seq data of testicular seminoma, and predicted ATBC target genes were obtained from public databases. Prognostic candidate genes were screened through differential expression analysis in seminoma datasets. Cox regression and machine learning algorithms were applied to the large TCGA-TGCT cohort to construct a robust prognostic risk model for patient stratification. Additional analyses included functional pathway enrichment, immune microenvironment assessment, single-cell gene expression profiling, key cell subtype identification, and clinical validation via immunohistochemistry and real-world clinical data.

**Results:**

PDK1 and OARD1 were confirmed as ATBC-associated prognostic genes for testicular seminoma. The established risk model showed moderate discriminatory capacity, with area under the curve (AUC) values for 1-, 2-, and 3-year survival all exceeding 0.6. Enriched pathways were mainly related to immunity and metabolism, and the high-risk group exhibited more active cell-mediated immune responses. Five cell subtypes were identified by scRNA-seq, among which B cells were recognized as the key cell population; dynamic expression changes of prognostic genes were observed during the differentiation of these key cells. Furthermore, protein levels of *PDK1* and *OARD1* were significantly higher in normal testicular tissues than in testicular seminoma tissues.

**Discussion:**

In conclusion, *PDK1* and *OARD1* are reliable prognostic biomarkers for testicular seminoma. This study provides an important theoretical basis for developing strategies to alleviate the adverse effects of ATBC on the male reproductive system.

## 1 Introduction

Acetyl tributyl citrate (ATBC), a citrate-based plasticizer commonly used in medical devices and food packaging materials, has emerged as a compound of oncological concern owing to its documented endocrine-disrupting properties (1–3). Epidemiological studies link urinary ATBC metabolites to a 27% increased risk of estrogen receptor-positive breast cancer, attributed to ERα coactivator recruitment and cell cycle dysregulation (2). In preclinical models, ATBC may promote glioma proliferation via AKT1, CASP3, HSP90AA1, ESR1, and MMP9 (2). These effects highlight its ability to perturb hormonal and inflammatory pathways critical for tissue homeostasis. Given the testis’ sensitivity to endocrine disruptors and AR signaling’s role in germ cell development, ATBC may influence seminoma pathogenesis. However, direct investigations into ATBC’s effects on seminoma cell proliferation, chemotherapeutic resistance, or metastatic behavior remain notably absent from the literature, thus representing a significant knowledge gap in our understanding of environmental risk factors contributing to this malignancy. Network toxicology offers a systems-level approach to map ATBC-protein interactions, identifying key pathways involved in its toxic effects (4, 5). By integrating databases like the Comparative Toxicogenomics Database (CTD), it helps to generate hypotheses regarding toxicity mechanisms beyond single targets, providing a holistic view of ATBC’s potential carcinogenic potential (6). Complementing network toxicology approaches, scRNA-seq addresses inherent limitations of bulk RNA-seq by resolving cellular heterogeneity at unprecedented resolution. It identifies rare cell subsets (e.g., cancer stem cells) and tracks dynamic state changes in the tumor microenvironment, enabling cell-type-specific analysis of gene expression (7, 8). In seminoma, this technology can pinpoint ATBC-responsive genes in tumor cells, immune infiltrates, or stromal cells, revealing cell-specific mechanisms of disease progression.

Testicular seminoma, a histologically distinct subtype of testicular germ cell tumors (TGCTs), arises from transformed germ cells and accounts for approximately 50% of all TGCT cases in adults (9, 10). Predominantly affecting males aged 15–34 years, seminoma represents the most common solid tumor in this age group. In 2023, an estimated 9,190 new testicular cancer cases and 470 deaths were reported in the United States, with mortality primarily attributed to metastatic disease and treatment resistance (11). Clinical presentation includes painless testicular masses, with advanced stages manifesting as retroperitoneal lymphadenopathy or pulmonary metastases. Current curative strategies integrate radical orchiectomy with adjuvant therapies: localized disease achieves >90% 5-year survival with radiotherapy or carboplatin, while metastatic disease requires platinum-based chemotherapy (12). Despite initial response rates of 70–80%, 30% of patients develop cisplatin resistance, leading to a dismal <50% 5-year survival in refractory cases (11). Furthermore, treatment-related toxicities, including secondary malignancies and cardiovascular complications, along with substantial economic burdens, emphasize the urgent need for molecular biomarkers to enable precision medicine approaches and optimize treatment selection. Although the pathogenesis of seminoma involves complex interactions between genetic predisposition, chromosomal aberrations (particularly isochromosome 12p), and potential environmental factors, reliable prognostic molecular markers that can guide clinical decision-making remain largely undefined.

This study aims to identify ATBC-associated prognostic signatures in seminoma using multi-omics methods. First, bulk RNA-seq data will be analyzed to screen survival-correlated genes and construct a machine-learning risk model, complemented by functional enrichment to characterize disrupted pathways and immune microenvironment differences (13). Second, scRNA-seq will map key genes to specific cell populations, elucidating ATBC’s cell-type-dependent effects (14). In this study, a two-tier strategy was employed: ATBC-associated targets were discovered exclusively in pure seminoma datasets to avoid tumor heterogeneity, while the prognostic risk model was constructed and validated in the broader TCGA-TGCT cohort to ensure adequate statistical power. These efforts seek to develop novel prognostic biomarkers for early risk stratification, uncover ATBC-mediated mechanisms in seminoma pathogenesis, and provide a translational framework for environmental chemical risk assessment in testicular cancer. Furthermore, by integrating clinicopathological feature analysis, this study aims to clarify the independent prognostic value and the specific functional patterns of PDK1 and OARD1 as differentiation-related markers in seminoma. Furthermore, by integrating clinicopathological feature analysis, this study aims to clarify the independent prognostic value and the specific functional patterns of PDK1 and OARD1 as differentiation-related markers in seminoma. By linking environmental exposure to molecular oncology, the research may inform targeted therapies, improving outcomes for patients and addressing unmet needs in TGCT management. This study also provides new methods and insights for understanding the impact of environmental pollutants on the occurrence and progression of malignant tumors, as well as for formulating public health strategies.

## 2 Materials and methods

### 2.1 Acquisition of data

The bulk RNA-seq expression matrix and survival data for testicular seminoma were obtained from the TCGA-TGCT dataset in the TCGA database (accessed on March 20, 2025). The TCGA-TGCT dataset consisted of 138 tumor tissue samples with progression-free interval (PFI) data (11). According to the official clinical annotations, these samples encompass the full spectrum of TGCTs, including 68 seminomas, 30 mixed germ cell tumors, 25 embryonal carcinomas, 11 teratomas, and 4 yolk sac tumors. To maintain sufficient sample size for machine learning algorithms, all 138 TGCT samples were retained and arbitrarily grouped into training and validation cohorts in a 5:5 ratio, with 69 samples in each cohort. Meanwhile, the GSE3218 and GSE228501 datasets were strictly limited to pure seminoma samples to ensure the specificity of target screening. Subsequently, the testicular seminoma-related bulk RNA-seq dataset GSE3218 and the scRNA-seq dataset GSE228501 were downloaded from the GEO database. The GSE3218 dataset, according to the GPL96 platform, included 12 testicular seminoma tumor tissue samples (tumor samples) and 6 control testis tissue samples (control samples) (15). Furthermore, 4 tumor tissue samples from testicular seminoma patients in the GSE228501 dataset, based on the GPL24676 platform, were included in this study (16).

The clinical specimens used in this study were provided by the Department of Pathology, First Hospital of Shanxi Medical University. This study was approved by the hospital’s ethics committee (No.: KYYJ-2025-115) and obtained informed consent from the patients. Patients with testicular germ cell carcinoma were selected as the case group, while normal testes removed due to testicular torsion were selected as the control group.

### 2.2 Prediction of the target genes for ATBC

To identify the target genes of ATBC, this compound was searched in the PubChem database to determine its standard structure and Simplified Molecular Input Line Entry System (SMILES) code. Using the retrieved information from PubChem, potential target genes 1 of ATBC were further explored in the CHEMBL database by searching with the keyword “ATBC”, restricting the search to “Homo sapiens”. Subsequently, the SMILES code of ATBC was submitted to the SwissTargetPrediction database (http://swisstargetprediction.ch/) to identify potential target genes 2. After merging and removing duplicates between potential target genes 1 and potential target genes 2, the target genes for ATBC were identified.

### 2.3 Differential expression analysis

We used the limma (v 3.54.0) package (17) to screen for Differentially Expressed Genes (DEGs) between the tumor and the control group in GSE3218 (|log_2_FC| > 0.5 and p < 0.05). Subsequently, we plotted a volcano plot using the ggplot2 (v 3.4.1) package, a heatmap using the ComplexHeatmap (v 2.18.0) package, and sorted the top 10 up- and down-regulated genes as per their |log_2_FC| values (from high to low).

### 2.4 Identification, enrichment analysis, and protein-protein interaction network of candidate genes

Based on DEGs, the VennDiagram (v 1.7.3) software package (18) was used to perform an intersection analysis between ATBC target genes and DEGs to screen for candidate genes. Subsequently, GO (p < 0.05) and KEGG analysis (adjusted p < 0.05) were utilized with the clusterProfiler (v 4.7.1.003) package (19) to investigate their biological functions. The top 5 pathways with the lowest p-values were selected and displayed for each entry in the GO enrichment results. Meanwhile, KEGG enrichment results were sorted by adjusted p-value (from smallest to largest) and presented as a bubble chart. Finally, candidate genes were submitted to the STRING database to gained protein-level interaction data (confidence > 0.40), and the Cytoscape (v 3.9.1) package was used to build a PPI network.

### 2.5 Selection of prognostic genes

Following this, the expression data of candidate genes were extracted and merged with the PFI information of 138 tumor samples. Within the training cohort from the TCGA-TGCT, to identify candidate genes related to the prognosis of tumor patients, these candidate genes were subjected to univariate Cox regression analysis by the survival (v 3.5.3) package (20) (p < 0.20), and then tested for the proportional hazards (PH) assumption using the cox.zph function (p > 0.05). Survival-related genes were then selected based on these results, and visualization was performed through a forest plot generated utilizing the forestplot (v 3.1.1) package. Subsequently, the LASSO regression analysis was conducted on these survival-related genes with 5-fold cross-validation, utilizing the glmnet (v 4.1.4) package (21). The optimal model fit was achieved when the lambda parameter was at its minimum, resulting in the selection of prognostic genes and their respective risk coefficients.

### 2.6 Construction and validation of risk model

Among the 69 tumor samples in the training cohort from TCGA-TGCT, a risk score was computed based on the expression levels of the previously identified prognostic genes and their corresponding risk coefficients. The formula was


$$risk\;score=\sum_{i=1}^{n}\left(coefi*Xi\right)$$

where *Xi* represented the expression level of each prognostic gene, and coefi denoted the risk coefficient of the corresponding gene. Under these circumstances, a risk model was constructed, and patients were partitioned into high-risk group (HRG) and low-risk group (LRG) according to the optimal cutoff value of the risk scores. Among the 69 tumor samples in the training cohort, the distribution of risk scores and survival status across different risk groups was illustrated. Kaplan-Meier (K-M) survival curves were then generated utilizing the survival (v 3.5.3) package, with the log-rank test applied to compare the PFI between the 2 risk groups (p < 0.05). Simultaneously, ROC curves were generated via the survivalROC (v 1.0.3.1) package (22) to evaluate the AUC values of the risk model for 1-, 2-, and 3-year survival (AUC > 0.60). To validate the risk model, a similar analysis was performed on the validation cohort from the TCGA-TGCT utilizing the same methodology.

### 2.7 Functional enrichment analysis

To explore the biological mechanisms underlying the differences between the 2 risk groups, differential gene expression analysis was performed utilizing the DESeq2 (v 1.38.0) package (23), and log_2_FC was calculated and sequenced in descending order. GSEA was carried out utilizing the clusterProfiler package (|NES| > 1 and p < 0.05), with the reference gene set “c2.cp.kegg.v7.4.symbols.gmt”. After that, the top 5 pathways were depicted via the enrichplot (v 1.18.4) package, ordered by p-values from lowest to highest.

To further investigate the pathway differences between the 2 risk groups, GSVA was executed with the GSVA (v 1.42.0) package (24), with the “c2.cp.kegg.v7.4.symbols.gmt” gene set from the MSigDB as the background. Enrichment marks for diverse pathways were computed, and variations in functional pathways between HRG and LRG were contrasted through the limma package (|t| > 2.0 and p < 0.05). The top 10 pathways were depicted in a bar chart, sorted by |t| values (ranked from highest to lowest).

### 2.8 Immune landscape analysis

Further examination focused on the comparative evaluation of immune cell infiltration levels within the immune microenvironments of the HRG and LRG in the training cohort. An in-depth analysis was performed using the ssGSEA algorithm from the GSVA (v 1.42.0) package to assess the infiltration levels of 28 distinct immune cells (25) in the HRG and LRG. The differences in immune cell infiltration between the HRG and LRG were evaluated to identify differentially infiltrated immune cells (p < 0.05), and the results were displayed appling a violin plot created with the ggplot2 package. Moreover, Spearman correlation analyses were conducted through the cor function from the stats (v 4.4.1) package (26).to investigate potential correlations between these differential immune infiltrating cells, as well as between prognostic genes and these disparate immune infiltrating cells (|cor| > 0.30 and p < 0.05). These correlations were then visualized using a heatmap generated by the ggcorrplot (v 3.5.1) package and a bubble diagram generated by the ggplot2 package, respectively.

### 2.9 Chromosomal localization and regulatory network of prognostic genes

We performed chromosomal localization analysis of prognostic genes via the RCircos (v 1.2.2) software. In order to ascertain the molecular regulatory mechanisms of the prognostic genes, the relationships between microRNAs (miRNAs) and prognostic genes were predicted utilizing the DIANA-microT database, along with the interactions between miRNAs and long non-coding RNAs (lncRNAs). Following this, a lncRNA-miRNA-mRNA regulatory network was built and graphically represented through the ggalluvial (v 0.12.5) package.

### 2.10 Molecular docking analyses and evaluation of toxicology

To clarify the specific interactions between the prognostic genes and ATBC, molecular docking was performed. The 3D model of ATBC was sourced from the PubChem database, while the protein conformations of the prognostic genes were extracted from the UniProt database. Molecular docking was carried out using the CB-Dock database. The binding energy, which indicated the interaction strength between the ligand and its target, was evaluated. A lower binding energy typically indicates a stronger interaction and higher binding affinity, with a threshold of ≤-5 kcal/mol generally regarded as indicative of favorable binding affinity. The PyMOL (v 2.5) software (27) was utilized to visualize the results.

To further assess the toxicity of ATBC, its chemical structure and canonical Simplified Molecular Input Line Entry System (SMILES) notation were retrieved from the ChEMBL database (https://www.ebi.ac.uk/chembl/). Toxicity predictions for ATBC were then conducted using the online platforms ProTox-3.0 (https://tox.charite.de/protox3), ADMET Lab 3.0 (admetlab3.scbdd.com), and ChemBlink (https://www.chemblink.com/).

To assess the dynamic stability of the receptor-ligand complexes, 100 ns molecular dynamics (MD) simulations were conducted using GROMACS software. The parameters assessed included Root Mean Square Deviation (RMSD), Root Mean Square Fluctuation (RMSF), the number of hydrogen bonds, and total system energy, which collectively evaluate the conformational stability and interaction reliability of the complexes in a simulated physiological environment. The computational analyses were strictly treated as predictive models for potential interactions rather than definitive proof of intracellular binding.

### 2.11 The scRNA-seq analysis

We analyzed the GSE228501 dataset using the Seurat (v 4.1.0) software package (28) to investigate the cellular mechanisms of testicular seminoma. First, low-quality data generated due to cell damage or during library preparation was filtered out through quality control (QC). The following criteria were applied (1): characteristic RNA quantity (number of genes per cell) <200 and >5000; (2) RNA count (gene expression level per cell) <30,000; (3) mitochondrial content percentage <30%. After filtering, the single-cell data were integrated using the IntegrateData function to remove the impact of varying sequencing depths across cells. Data normalization was carried out through the NormalizeData function. The FindVariableFeatures function and vst method were employed to identify the top 2,000 highly variable genes (HVGs), with the top 10 genes exhibiting the most pronounced expression fluctuations highlighted in the visual output from the LabelPoints function.

Following this, principal component analysis (PCA) was performed on the selected HVGs, using the top 50 PCs to extract the main variant features in the high-dimensional data through the runPCA function. The JackStraw (p < 0.05) method was used to evaluate the contribution of the top 50 principal components, and the ElbowPlot function was used to draw an elbow plot to observe their contribution to the variation. Next, cell clustering analysis was carried out on cells post-dimension reduction through the FindNeighbors and FindClusters functions (resolution set to 0.1). The cell clusters in the scRNA-seq data were then visualized by Uniform Manifold Approximation and Projection (UMAP). Finally, according to the marker genes pinpointed by the literature (16), the cell clusters were annotated.

### 2.12 Analysis of cell functional characterization and identification of key cells

We executed functional enrichment analysis of annotated cell types via the analyze_scClusters function of the ReactomeGSA (v 1.16.1) package (29) (p < 0.05), and the top 15 pathways (ranked in descending order based on the difference in the values of the highest and lowest pathway enrichment scores) were selected and visualized using a heatmap. Through further analysis, immune cell types with highly expressed genes and significant differences in the immune microenvironment were identified as key cells.

### 2.13 Cell communication and pseudo-time analysis of key cells

We conducted intercellular communication analysis via the CellChat (v 1.5.0) software package (30) to infer complex interactions between cell types, focusing on key cell types. Receptor-ligand interactions were visualized using a bubble chart (p < 0.01), illustrating the signaling exchange between the annotated cell types.

To delve further into the heterogeneity of the key cells, they were subdivided into distinct subclusters through embedding and clustering analysis. Moreover, the monocle2 (v 2.24.1) package (31) was utilized to carry out pseudo-time analysis, with a focus on the temporal changes in the expression of genes.

### 2.14 Expression analysis of prognostic genes

The expression of prognostic genes was further verified through immunohistochemistry at the Pathology Department of Shanxi Medical University First Hospital, and the resultant data were statistically analyzed by Graphpad Prism (v 10.0) software.

## 3 Results

### 3.1 Identification and enrichment analysis of 20 candidate genes

At the initiation of this study, 40 potential target genes 1 of ATBC were identified using the CHEMBL database, and the SwissTargetPrediction database facilitated the identification of 13 potential target genes 2 corresponding to ATBC (**Supplementary Table 1**). Subsequently, a merged and deduplicated analysis was conducted between potential target genes 1 and potential target genes 2, resulting in a total of 53 target genes for ATBC (**Supplementary Table 1**).

**Figure 1:**
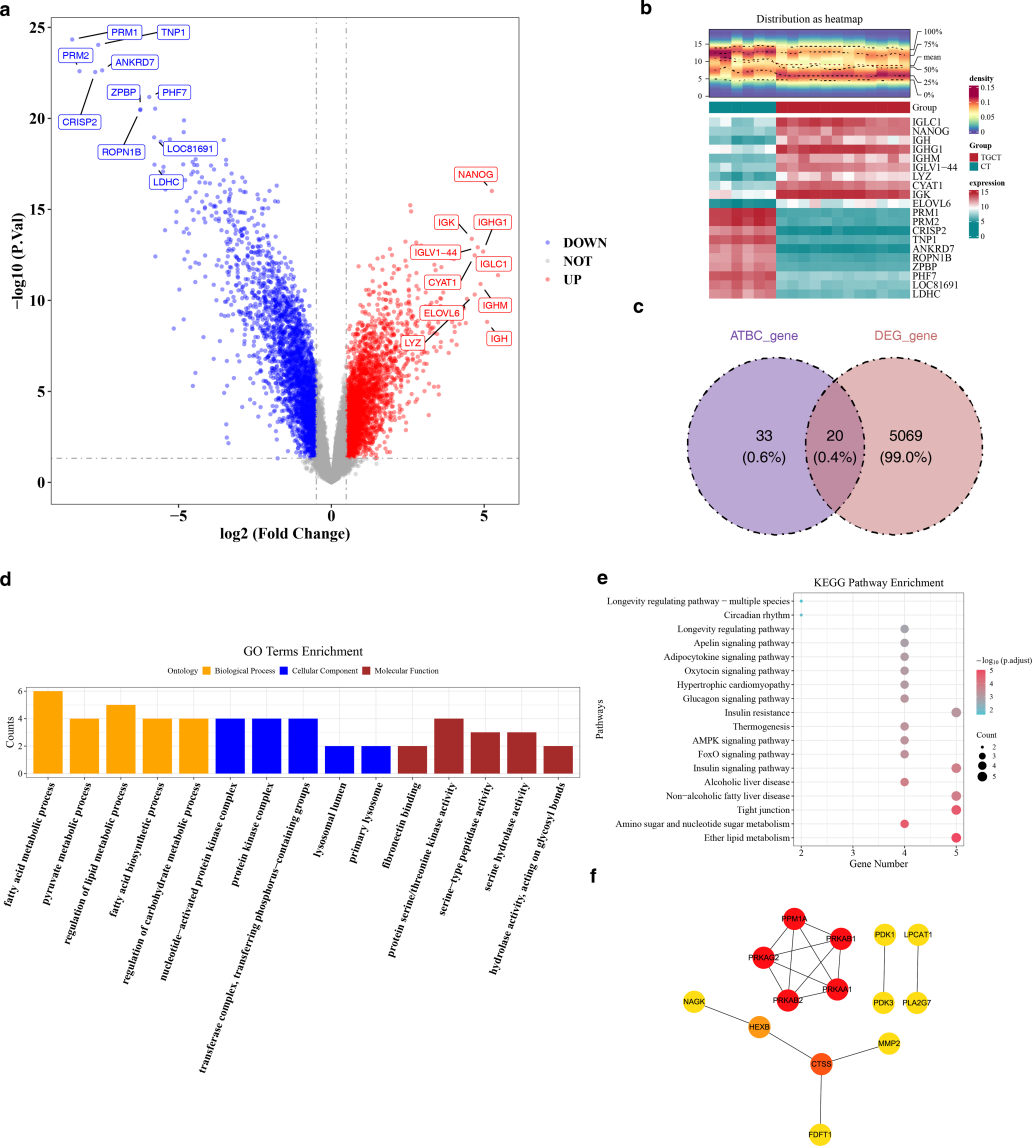
Screening of differentially expressed genes influencing TGCT by ATBC. **(a)** Volcano plot of differentially expressed genes between the TGCT group and the control group. Red represents upregulation, and blue represents downregulation. **(b)** Heatmap of differentially expressed genes between the TGCT group and the control group. Red indicates high expression, and turquoise indicates low expression. The darker the color, the greater the expression level. **(c)** Venn diagram of the intersection of differentially expressed genes. **(d)** GO enrichment results. Yellow represents BP, blue represents CC, and red represents MF. **(e)** Bubble plot of the KEGG enrichment analysis results. **(f)** PPI network.

Following the differential expression analysis executed in the GSE3218 dataset, 5,089 DEGs were identified between tumor and control samples, with 2,595 genes up- and 2,494 genes down-regulated in tumor (|log_2_FC| > 0.5 and p < 0.05) (**Figures 1a, b**). Subsequently, the intersection of 53 target genes for ATBC and 5,089 DEGs yielded 20 candidate genes (**Figure 1c**).

Based on the above results, GO analysis identified a total of 390 significantly different terms (p < 0.05), covering 333 BP, 15 CC, and 42 MF (**Supplementary Table 2**). In the GO, the candidate genes were mainly associated with functions such as “pyruvate metabolic process” (BP), “protein kinase complex” (CC), and “fibronectin binding” (MF) (p < 0.05) (**Figure 1d**; **Supplementary Table 2**). Moreover, 18 KEGG (adj. p < 0.05) pathways were identified, including “circadian rhythm”, “longevity regulating pathway”, and “apelin signaling pathway” (**Figure 1e**; **Supplementary Table 2**). After removing 6 separate proteins, the remaining 14 genes were displayed in the PPI network, with such genes as PRKAB1, PRKAB2, and PPM1A presenting intense interactions with other proteins (**Figure 1f**).

### 3.2 Selection of *PDK1* and *OARD1* as prognostic genes

**Figure 2:**
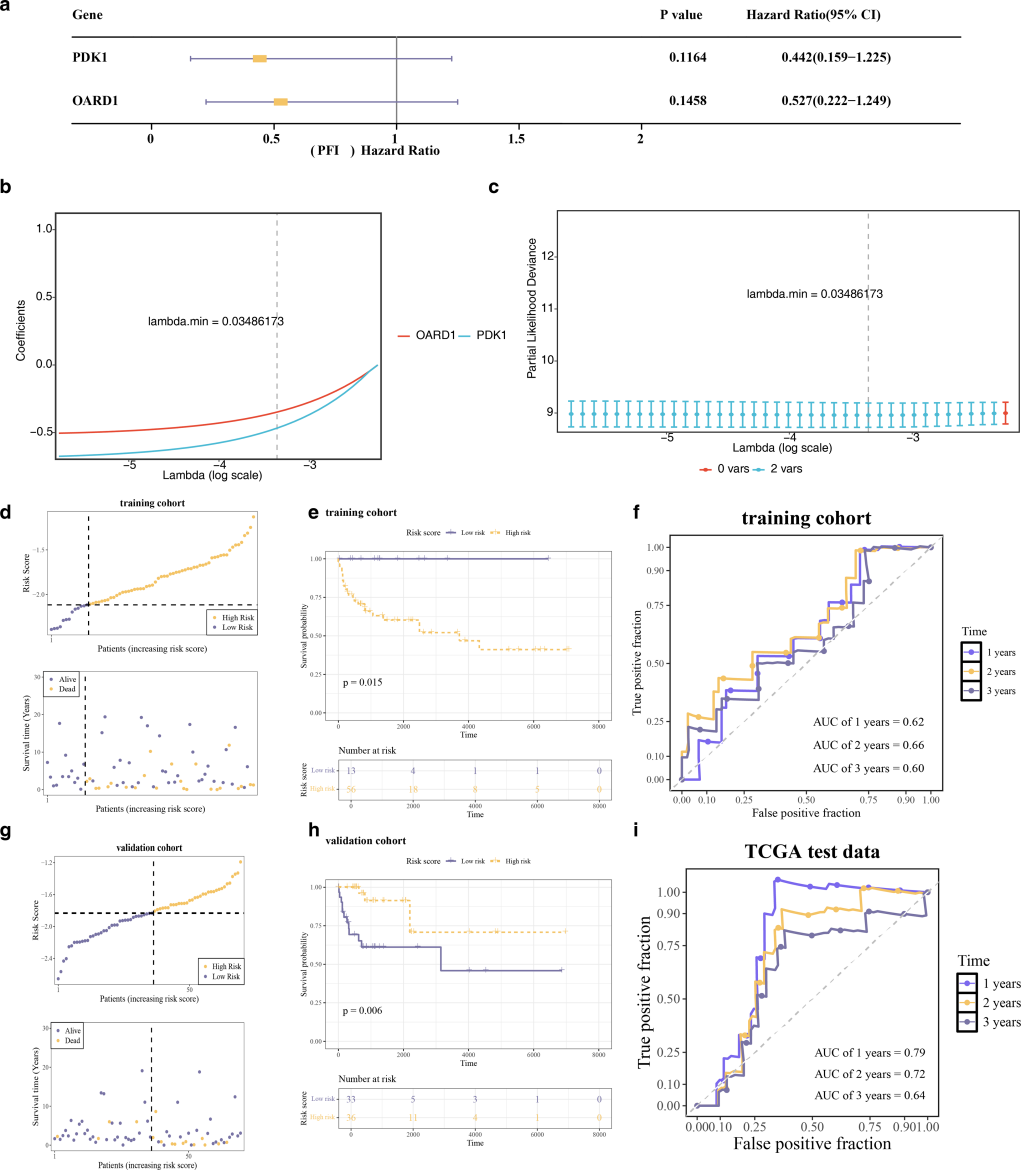
Machine learning analysis. **(a)** Univariate cox forest plot of prognosis-related genes. The yellow dots represent the Hazard Ratio (HR) values. Genes with HR>1 are risk genes, and genes with HR<1 are protective genes. **(b)** Results of LASSO regression analysis. The abscissa is the logarithm of lambdas, and the ordinate is the variable coefficient. **(c)** Error plot of cross-validation of LASSO regression analysis. The abscissa is the logarithm of lambdas, and the ordinate is the model error. **(d)** Risk curve (upper) and survival status distribution (lower) of patient samples in the training set. In the upper figure, the yellow dots represent high-risk patients, and the purple dots represent low-risk patients. In the lower figure, the yellow dots represent deceased patients, and the purple dots represent surviving patients. **(e)** KM curve of HRG and LRG in the training set. The yellow dots represent high - risk patients, and the purple dots represent low-risk patients. **(f)** Heatmap of prognostic gene expression in the training set. Yellow represents the HRG, and purple represents the LRG. **(g)** Risk curve (upper) and survival status distribution (lower) of patient samples in the validation set. In the upper figure, the yellow dots represent high - risk patients, and the purple dots represent low - risk patients. In the lower figure, the yellow dots represent deceased patients, and the purple dots represent surviving patients. **(h)** KM curve of HRG and LRG in the validation set. The yellow dots represent high - risk patients, and the purple dots represent low-risk patients. **(i)** Heatmap of prognostic gene expression in the validation set. Yellow represents the HRG and purple represents the LRG.

Subsequently, the 20 candidate genes were subjected to univariate Cox regression analysis (p < 0.20), followed by the PH assumption test (p > 0.05) in the training cohort from the TCGA-TGCT, resulting in the identification of 2 survival-related genes (**Figure 2a**; **Supplementary Table 3**). Further screening using the LASSO algorithm with 5-fold cross-validation revealed 2 prognostic genes at lambda.min = 0.03486173, namely *PDK1* and *OARD1* (**Figures 2b, c**). These results further highlighted the prognostic value of *PDK1* and *OARD1* in testicular seminoma.

### 3.3 Robust predictive ability of the risk model

Capitalizing on the gene expression data of prognostic genes and their corresponding risk coefficients, a risk score equation was formulated: risk score = *PDK1* × (-0.4667375) + *OARD1* × (-0.3480846), and a risk model was then constructed, offering a quantitative tool for prognostication. Based on the optimal cutoff values (-2.115006 for the training cohort and -1.832612 for the validation cohort), tumor samples were stratified into 2 risk groups within both the training (n = 69, high = 56, low = 13) and validation (n = 69, high = 33, low = 36) cohorts.

Following that, the distribution of risk scores and the distribution of survival status across distinct risk groups in the training cohort were depicted in **Figure 2d**, demonstrating that the number of deaths increased with rising risk scores. The survival differences between patients in the different risk groups were further assessed, and patients in HRG exhibited poorer survival rates compared to those in the LRG (p = 0.015) (**Figure 2e**). ROC curves demonstrated strong predictive ability for the risk model, with AUC values of 0.62, 0.66, and 0.60 for 1-, 2-, and 3-year survival in the training cohort, respectively (**Figure 2f**). Besides, the validation of the risk model in the validation cohort from the TCGA-TGCT confirmed its robustness (**Figures 2g–i**), with AUC values exceeding 0.60 for 1-, 2-, and 3-year survival, highlighting the model’s consistent prognostic power.

### 3.4 Differential expression of PDK1 and OARD1 across clinicopathological characteristics

To directly address the specific roles of PDK1 and OARD1 in seminoma progression, differential expression analyses of these genes were conducted across distinct clinicopathological subgroups, including age, T stage, N stage, M stage, overall stage, and treatment status. Notably, the expression levels of both PDK1 and OARD1 exhibited no significant differences across these clinical subgroups (all p > 0.05) (**Supplementary Figure 1****).** These findings indicated that rather than acting as classical cancer-promoting drivers that fluctuate with tumor macroscopic progression (such as staging or metastasis), the low expression of PDK1 and OARD1 serves as an intrinsic, independent molecular feature accompanying the progression of seminoma.

### 3.5 Exploration of the potential mechanisms

GSEA was conducted to explore potential mechanisms underlying the distinct risk groups, revealing that 37 KEGG pathways were substantially enriched (p < 0.05 and |NES| > 1) (**Supplementary Table 4**). Specifically, among the top 5 pathways, “neuroactive ligand receptor interaction”, “complement and coagulation cascades”, “maturity onset diabetes of the young”, “steroid hormone biosynthesis”, and “focal adhesion” were all predominantly enriched in the HRG (**Supplementary Figure 2A**). Following the GSVA conducted between HRG and LRG, a total of 55 significant differential pathways were identified, with 41 activated and 14 inhibited in HRG samples (**Supplementary Table 5**). The activated pathways included “steroid hormone biosynthesis”, “neuroactive ligand receptor interaction”, and “histidine metabolism” (**Supplementary Figure 2B**). The inhibited pathways comprised “mismatch repair”, “ubiquitin mediated proteolysis”, and “homologous recombination” (**Supplementary Figure 2B**). These pathways might be critical factors in influencing the clinical outcomes of testicular seminoma patients. Understanding these mechanisms could help in developing new therapeutic strategies and identifying potential targets for future research.

### 3.6 Dissection of the immunological landscapes across risk groups

**Figure 3:**
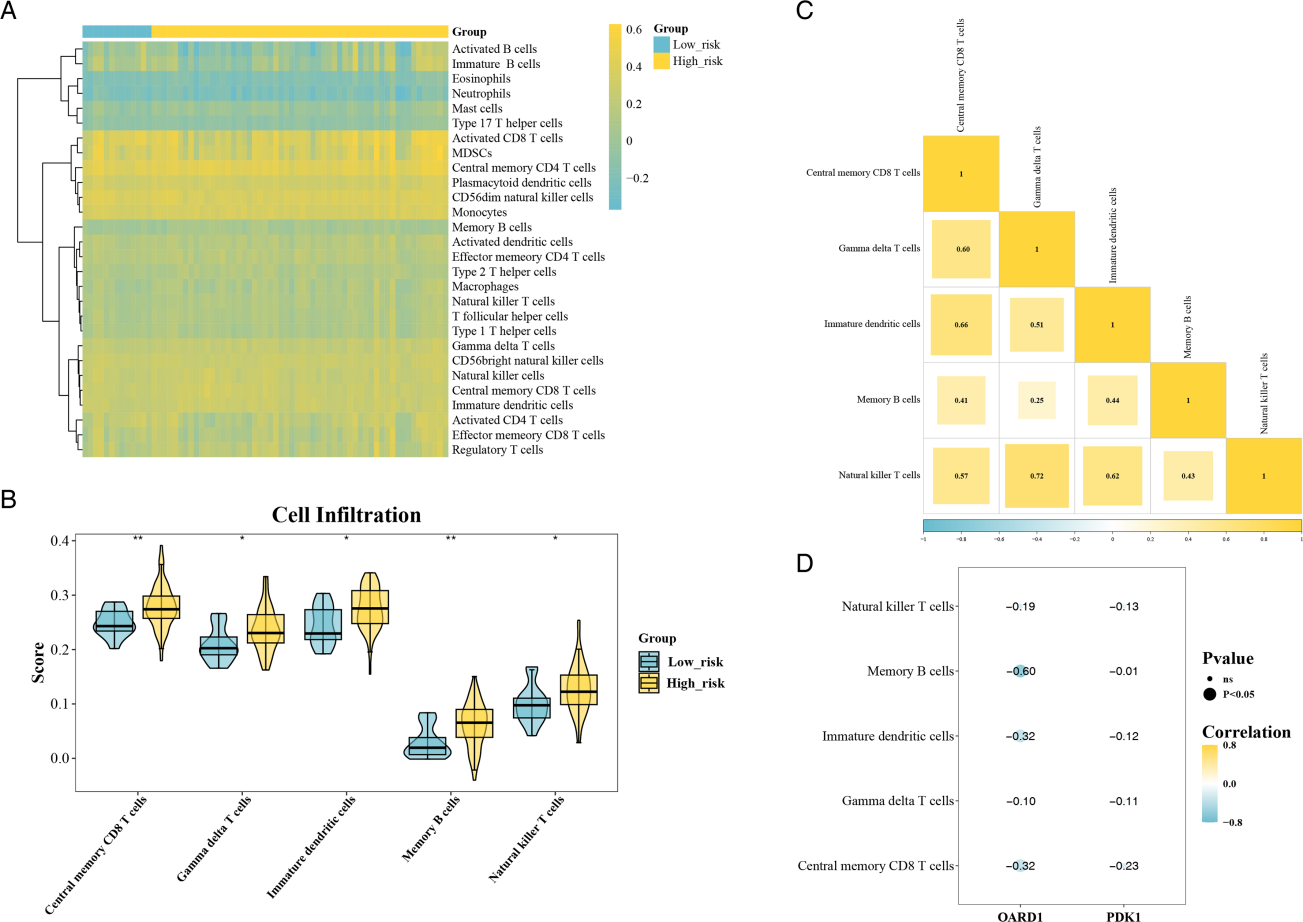
Immune cell infiltration analysis. **(A)** Immune cell abundance. The upper part of the heatmap is the grouping bar, with yellow indicating the HRG and blue indicating the LRG. The higher the level of immune infiltration, the more the color approaches yellow, and the lower it is, the more the color approaches blue. **(B)** Differentially infiltrated immune cells between HRG and LRG samples. Blue represents low-risk samples, and yellow represents high-risk samples. **(C)** Differentially infiltrated immune cells between HRG and LRG samples. Blue represents low-risk samples, and yellow represents high-risk samples. **(D)** Heatmap of the correlation between prognostic genes and differentially infiltrated immune cells. Blue represents a negative correlation, and yellow represents a positive correlation. * p < 0.05, ** p < 0.01.

Subsequently, a heatmap was generated to display the ssGSEA scores of 28 immune infiltrating cells in samples from the HRG and LRG within the training cohort from the TCGA-TGCT (**Figure 3A**). A violin plot further visualized these differences between the 2 risk groups, revealing significant disparities in 5 types of immune infiltrating cells (p < 0.05) (**Figure 3B**). Notably, these 5 differential immune infiltrating cells exhibited significantly higher ssGSEA scores in the HRG (p < 0.05), suggesting a more active cell-mediated immune response in the HRG, which might be linked to tumor immune evasion mechanisms and the complex interactions.

Furthermore, correlation analysis demonstrated that most of the differential immune infiltrating cells were significantly positively correlated with each other (|cor| > 0.30 and p < 0.05), with the strongest positive correlation observed between natural killer T cells and gamma delta T cells (cor = 0.72 and p < 0.0001) (**Figure 3C**; **Supplementary Table 6**). Regarding prognostic genes, *OARD1* exhibited the highest significant negative correlation with memory B cells (cor = -0.60 and p < 0.0001) (**Figure 3D**; **Supplementary Table 7**). No significant correlation was found between *PDK1* and these differential immune infiltrating cells (|cor| < 0.30) (**Figure 3D**; **Supplementary Table 7**). These findings provided important insights into the immune landscape of different risk groups and highlighted potential targets for future research and therapeutic interventions.

### 3.7 Unraveling the chromosomal localization and potential molecular regulatory mechanisms of prognostic genes

Chromosomal localization analysis revealed that *PDK1* was located on chromosome 2, while *OARD1* was located on chromosome 6 (**Supplementary Figure 3A**). Further prediction analysis using the DIANA-microT database revealed that 12 miRNAs were associated with *PDK1* and 30 miRNAs with *OARD1* (**Supplementary Table 8**). Meanwhile, only 3 miRNAs (hsa-miR-106a-5p, hsa-miR-3150b-3p, and hsa-miR-3163) were shown to be associated with the regulation of lncRNAs, resulting in the identification of 4 lncRNAs (AC021078.1, AL355075.4, NORAD, and MALAT1). Based on these interaction pairs, a lncRNA-miRNA-mRNA regulatory network was built (**Supplementary Figure 3B**), revealing complex interaction relationships. For instance, MALAT1 was regulated by hsa-miR-3163, which in turn regulated *PDK1*. These results offered valuable insights for investigating the roles of these interactions in the mechanisms of testicular seminoma.

**Figure 4:**
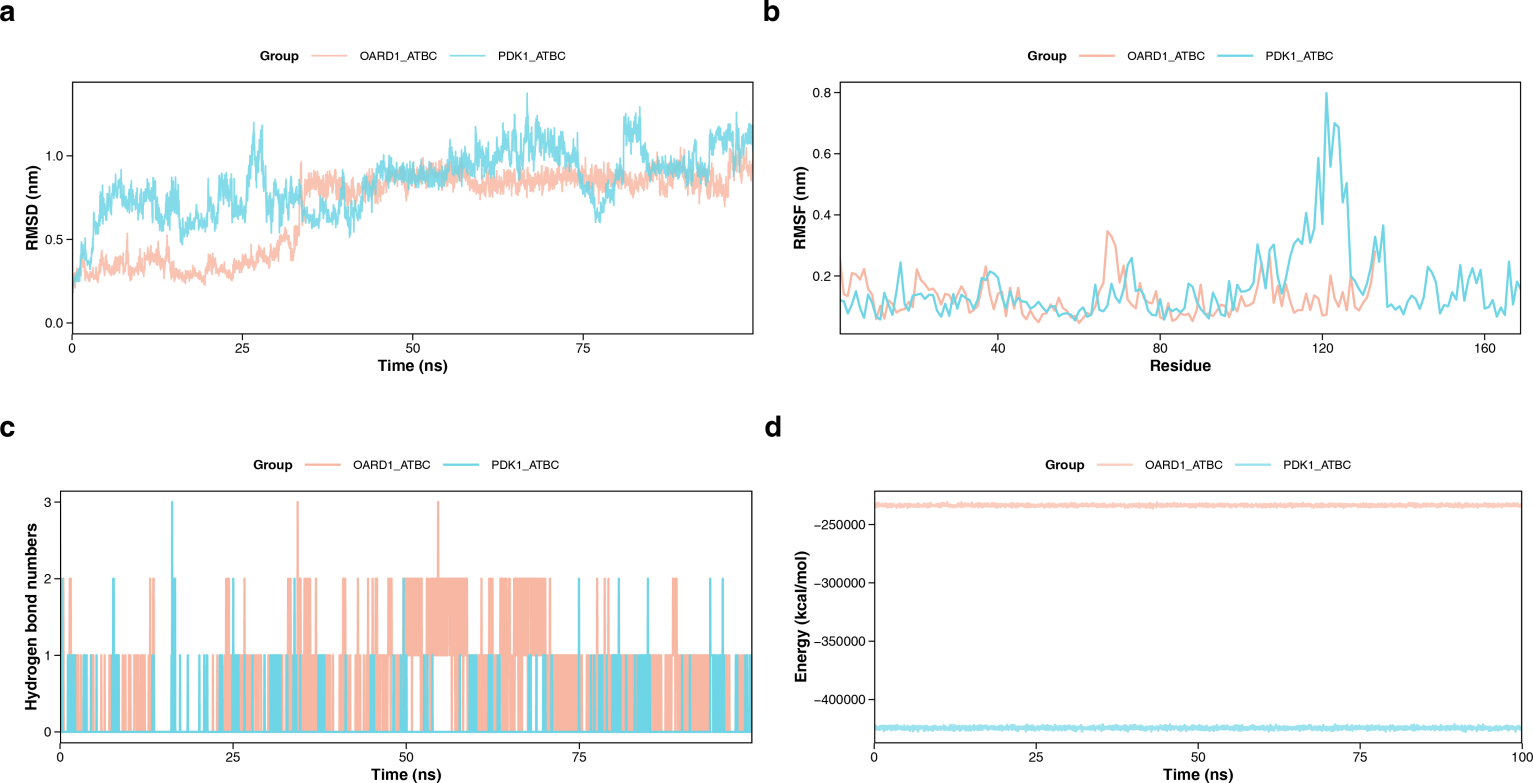
Molecular dynamics (MD) simulation analysis of ATBC in complex with PDK1 and OARD1 over 100 ns. **(A)** Root Mean Square Deviation (RMSD) trajectories of the complexes, showing equilibration and stable plateaus after approximately 40 ns. **(B)** Root Mean Square Fluctuation (RMSF) profiles of the protein residues, indicating high overall conformational rigidity with fluctuations predominantly below 1.0 nm. **(C)** The number of hydrogen bonds formed between ATBC and the target proteins over time, demonstrating a dynamically stable interaction interface. **(D)** Total system energy of the complexes throughout the simulation, reflecting excellent thermodynamic stability. Light red lines represent the OARD1-ATBC complex, and light blue lines represent the PDK1-ATBC complex.

To further validate the dynamic stability of the docking models, 100 ns MD simulations were executed (**Figure 4**). The RMSD trajectories of both the OARD1-ATBC and PDK1-ATBC complexes reached a plateau after approximately 40 ns, indicating that the systems entered a stable equilibrium state. The RMSF values for the vast majority of residues remained within the 0 to 1 nm range throughout the 100 ns duration, revealing high overall conformational stability with minimal structural unfolding. Furthermore, dynamic hydrogen bond analysis showed persistent and periodic fluctuations in hydrogen bond networks, reflecting a stable yet elastic interaction at the binding interface. The total energy of both systems remained exceptionally flat and stable throughout the simulation. Together, these computational findings reliably suggest a potential structural basis for ATBC’s interaction with PDK1 and OARD1, forming a supplementary hypothesis for its toxicological mechanisms.

### 3.8 Elucidating the interaction between prognostic genes and ATBC

**Figure 5:**
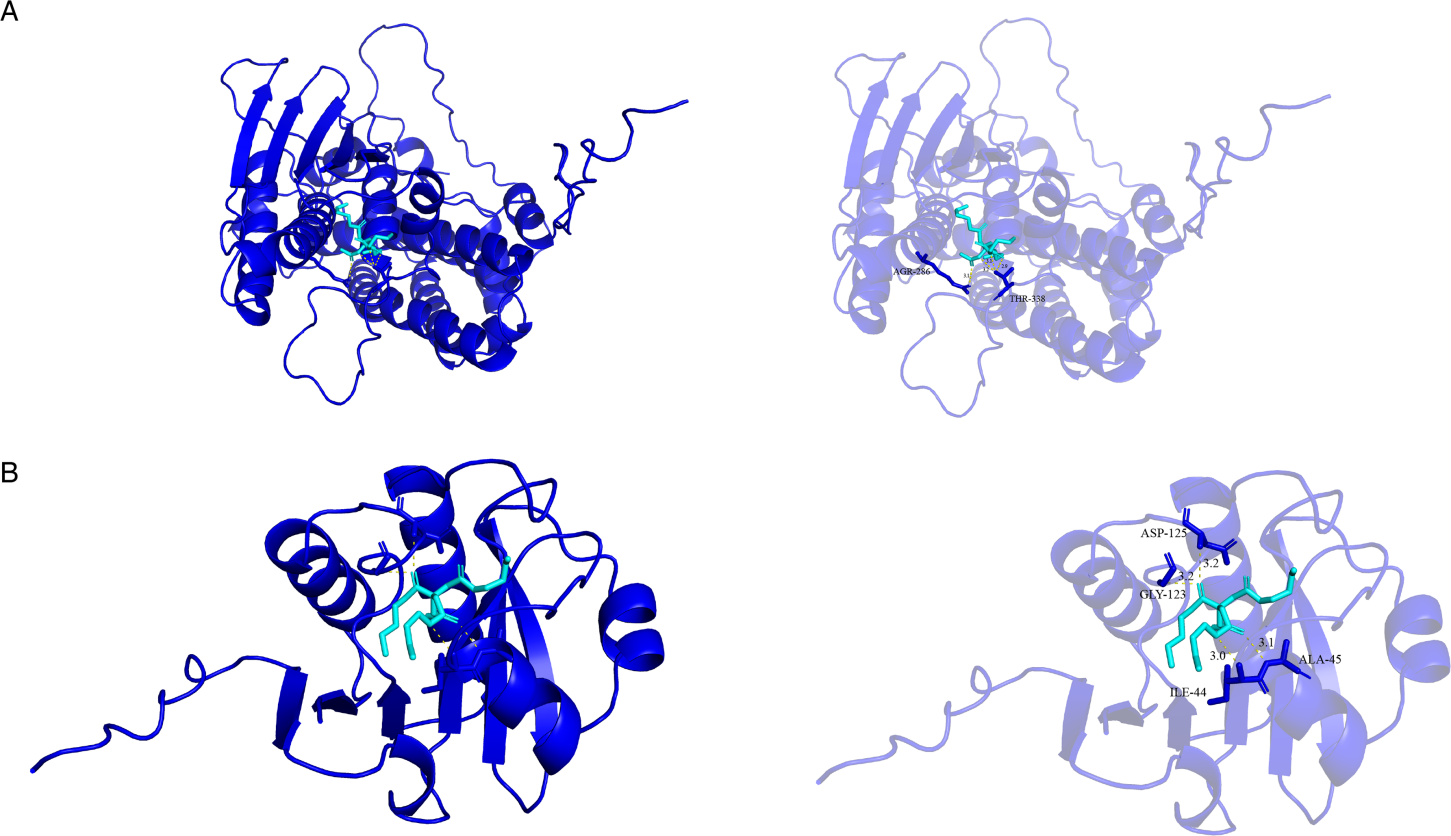
Molecular docking analysis. **(A)** Molecular docking sites of ATBC and the protein encoded by *PDK1*. **(B)** Molecular docking sites of ATBC and the protein encoded by *OARD1*. In the figure, the cyan-blue part represents the drug ligand. The dark - blue helix represents the protein molecule. The drug ligand acts on the protein molecule through hydrogen bonds, and the yellow markings clearly indicate the positions of the hydrogen bonds.

To gain deeper insights into the specific interactions between prognostic genes and ATBC, molecular docking analysis was performed. The results demonstrated that the prognostic genes exhibited strong binding affinities with ATBC (**Table 1**), with the highest binding affinity observed between *PDK1* and ATBC. Specifically, *PDK1* showed a binding energy of -6.5 kcal/mol with ATBC, with hydrogen bonds formed by the ARG-286 and THR-338 residues (**Figure 5A**). For *OARD1*, the binding energy with ATBC was -5.5 kcal/mol, with the GLY-123, ASP-125, ILE-44, and ALA-45 residues forming hydrogen bonds (**Figure 5B**). These results elucidated the robust interactions between the prognostic genes and ATBC, emphasizing their potential functional roles in further biological mechanisms.

**Table 1:** Binding energies of prognostic genes and ATBC.

Gene	ATBC	Binding energy (kcal/mol)
*PDK1*	ATBC	-6.5
*OARD1*	ATBC	-5.5

The toxicity of ATBC was evaluated using three different platforms, which revealed consistent findings regarding its potential hazards. ProTox-3.0 indicated that ATBC exhibited carcinogenic potential with a probability of 0.61, but no significant toxicity was observed in other organs (**Supplementary Table 9**). ADMET Lab 3.0 results also confirmed its carcinogenicity, with a lower probability of 0.255, while highlighting that ATBC caused noticeable skin and eye irritation, with no significant toxicity to other organs (**Supplementary Table 10**). ChemBlink’s assessment further supported the carcinogenicity of ATBC and reported similar skin and eye irritation, in addition to indicating that ATBC posed a long-term hazard to aquatic environments (**Supplementary Table 11**). Overall, these analyses suggested that ATBC was carcinogenic, irritated the skin and eyes, and presented an environmental risk to aquatic life, without significant toxicity to other organs.

### 3.9 Annotation of 5 cell types

Following a comprehensive bulk RNA-seq analysis, scRNA-seq analysis was conducted on the GSE228501 dataset to further explore the underlying mechanisms of testicular seminoma. Originally, visuals of nFeature RNA, nCount RNA, and mitochondrial composition percentage before and after QC were output (**Supplementary Figures 4A, B**). Altogether 16,077 cells and 24,232 genes were kept after filtering out ineligible cells and genes. Standard data processing was followed by the selection of 2,000 HVGs, and the 10 genes with the most variation in expression levels were highlighted (**Supplementary Figure 4C**). Following PCA, the top 30 were chosen for subsequent analysis (**Supplementary Figures 5A, B**). Subsequent clustering analysis indicated 9 different cell clusters (**Supplementary Figure 5C**), which were further annotated to identify 5 cell: seminoma cells, endothelial cells, and macrophages, etc. (**Supplementary Figure 5D**). Meanwhile, the dotplot demonstrated the high specificity of marker genes, affirming the precision of the annotations (**Supplementary Figure 5E**).

### 3.10 Comprehensive cellular biological functions and B cells as key cells

**Figure 6:**
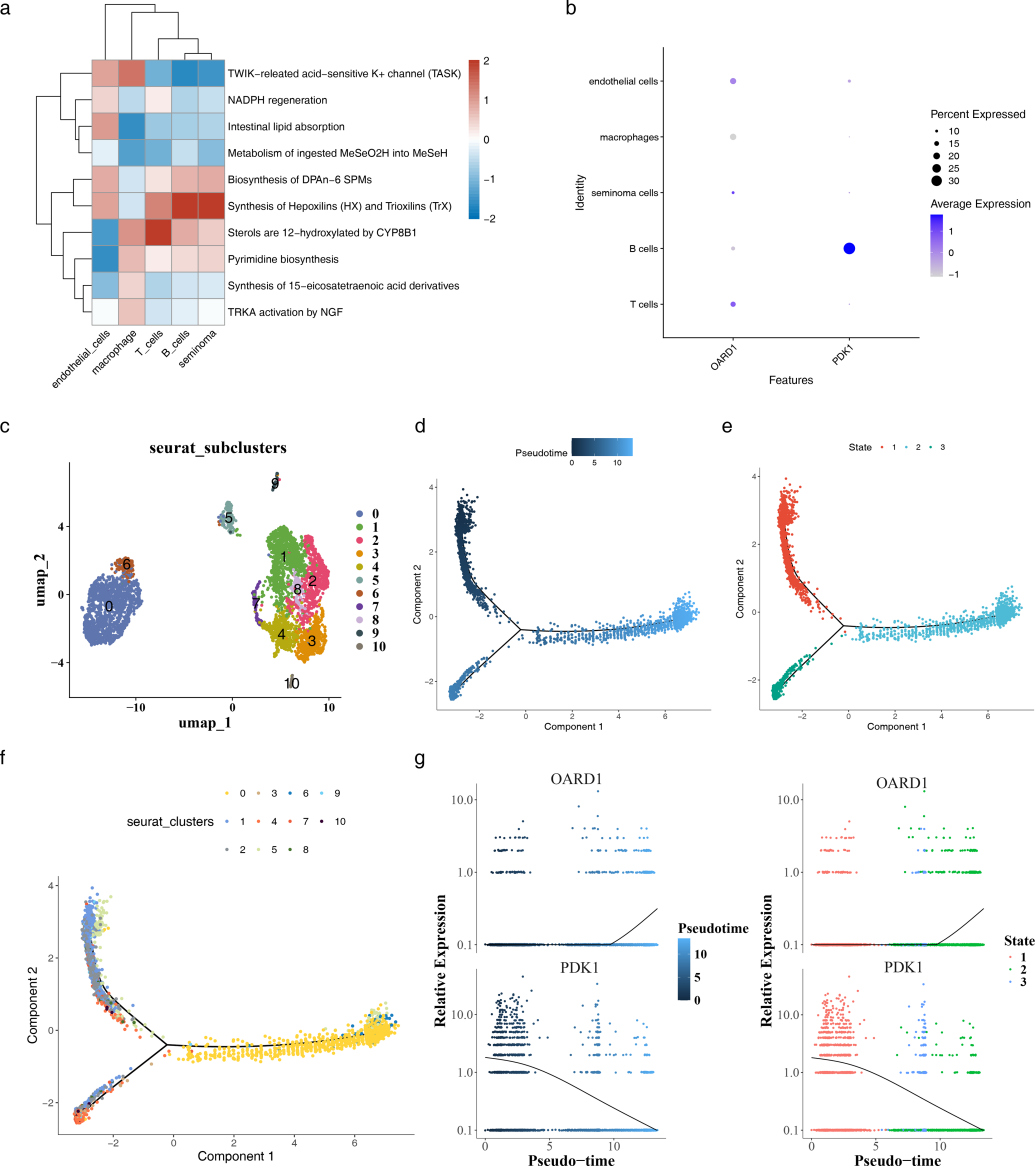
Cell clustering annotation and pseudo temporal analysis. **(a)** Functional pathway enrichment analysis of annotated cells (TOP 10). Blue represents low enrichment and red represents high enrichment. **(b)** Correlation bubble plot of *PDK1* and *OARD1* with different types of cells. The larger the dot, the higher the proportion of the quantity, and the darker the color of the dot, the higher the expression level. **(c)** UMAP cell distribution dimensionality reduction plot, with different colors representing different cell clusters. **(d, e)** Pseudo temporal analysis cell trajectory plot. **(f)** Distribution of B cell differentiation stages across distinct subclusters. **(g)** Dynamic expression patterns of prognostic genes along pseudotime trajectory.

Following that, those 5 cell types were enriched in pathways such as “NADPH regeneration”, “TNFR1-mediated ceramide production”, and “pyrophosphate hydrolysis” (p < 0.05) (**Figure 6a**; **Supplementary Table 12**). Then, the expression of prognostic genes across the 5 cell types was visualized in a bubble chart (**Figure 6b**). Notably, both prognostic genes were highly expressed in B cells, suggesting that B cells could be considered as key cells. Significant differences in memory B cells were observed in the immune microenvironment. The identification of B cells as key cells underscored their potential role in testicular seminoma development and highlighted the need for further investigation into their functional mechanisms.

### 3.11 Specific communications of cell types

Cell-cell communication analysis in 5 cell types revealed that, compared with other cell types, the strongest interactions were noted between B cells and macrophages in testicular seminoma samples (**Supplementary Figures 6A, B**). Furthermore, the highest interaction was seen between seminoma cells and macrophages, with the ligand-receptor interplay involving MIF-(CD74+CXCR4) (p < 0.01) (**Supplementary Figure 6C**). Additionally, the MIF-(CD74+CXCR4) signaling axis was identified as a key intermediary in the interactions between B cells and macrophages, while the TNFSF13B-TNFRSF17 signaling axis was crucial for interactions between macrophages and B cells (p < 0.01) (**Supplementary Figure 6C**). This highlighted the potential for modulating immune cell interactions in testicular seminoma, opening the door for novel therapeutic strategies aimed at enhancing immune responses or inhibiting tumor progression.

### 3.12 Pseudo-time trajectory deduction of B cells

Dimension reduction and cluster analysis divided B cells into 11 subpopulations (resolution = 0.4) (**Figure 6c**). Then, the beginning of the temporal trajectory of B cells was established, demonstrating they experienced maturation in their development (**Figure 6d**). B cell trajectories were deduced through analysis and classified into 3 states (**Figure 6e**). Subcluster was found to be in the late stages of cell differentiation, while subcluster 4 and subcluster 7 were predominantly observed in the early and mid stages of differentiation (**Figure 6f**).

Additionally, the expression levels of prognostic genes were also observed at different developmental stages of B cells (**Figure 6g**). Specifically, *PDK1* exhibited a gradual decline along the pseudo-time trajectory, with higher expression in state 1; while *OARD1* was primarily expressed at the late stages of differentiation, showing higher expression in state 2. These results suggested that the expression levels of prognostic genes were characterized by dynamic changes during the differentiation process of B cells.

### 3.13 Verification of prognostic genes expression

**Figure 7:**
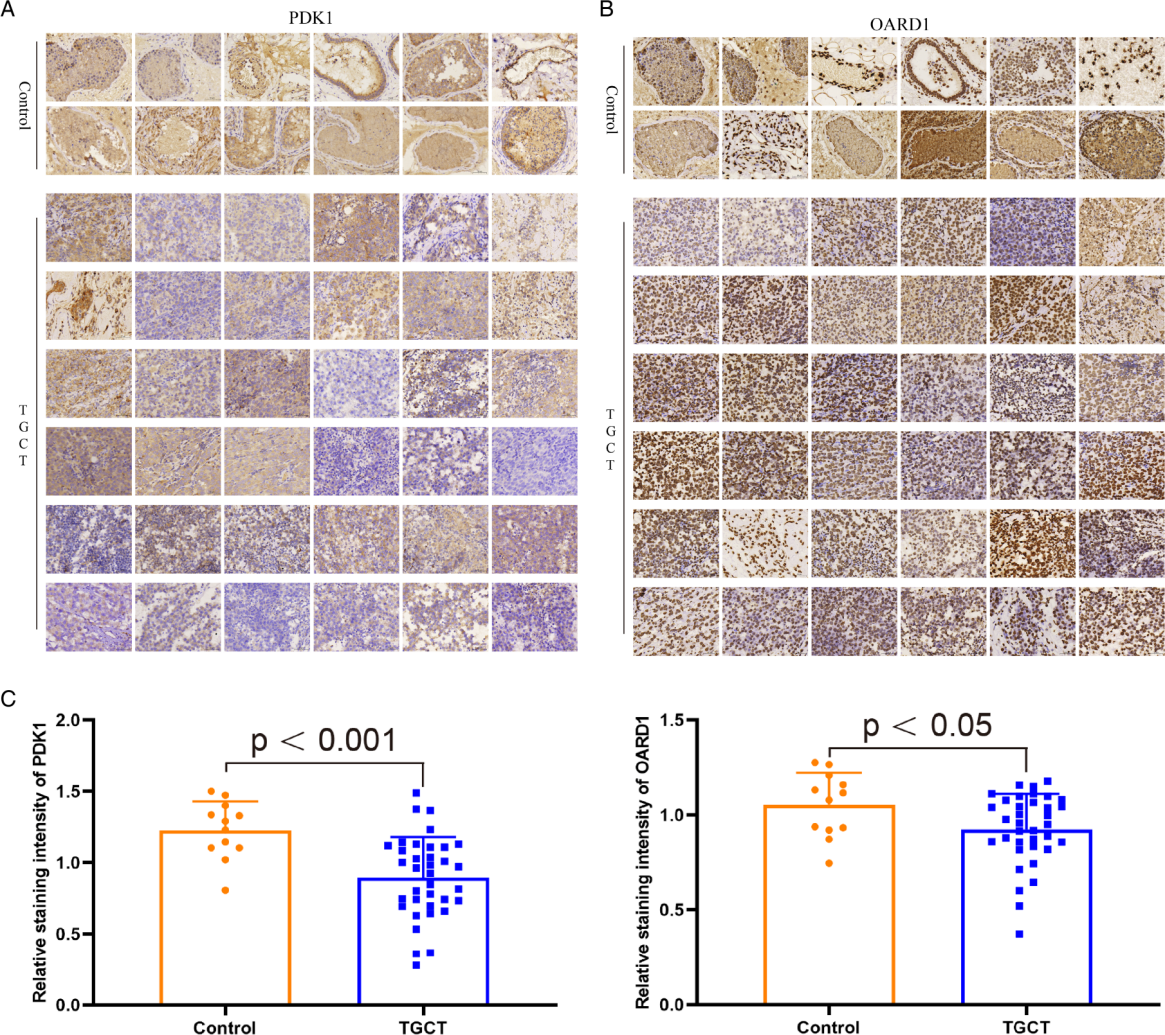
Immunohistochemical analysis of genes *PDK1* and *OARD1* Immunohistochemical expression of proteins *PDK1*. **(A)** and *OARD1* **(B)** in normal tissues and TGCT. **(C)** Immunohistochemical quantification of *PDK1* and *OARD1* protein expression in normal tissues and TGCT.

The demographic data of clinical data for the control group and case group patients have been provided in **Table 2**. Further verification of the expression of prognostic genes *PDK1* and *OARD1* in normal testes and TGCT was conducted through immunohistochemistry. Immunohistochemical staining showed the protein expression status of *PDK1* and *OARD1* in normal testes and TGCT, as shown in **Figures 7A, B**. The results showed that the protein expression levels of *PDK1* and *OARD1* were significantly decreased in testicular seminomas compared to the control group (**Figure 7C**).

**Table 2:** The demographic data of clinical data for the control and TGCT.

Category	Control	TGCT
NO.	36	12
Age		
>50	6	0
≤50	30	12

## 4 Discussion

ATBC, as a commonly used plasticizer characterized by high leaching potential and endocrine-disrupting properties, has received increasing concern from the scientific community. Structurally and functionally, ATBC shares similarities with phthalate compounds in terms of plasticizing properties and endocrine-disrupting potential. Studies have demonstrated that phthalate esters function as endocrine disruptors that interfere with hormonal homeostasis, and these compounds have been associated with increased risk of male genitourinary tumors (32, 33). Although studies on the association between ATBC and TGCT are limited, the carcinogenic potential of ATBC may be a risk factor for testicular tumors. This study is mainly based on a public database. By integrating ATBC target prediction and DEGs, we identified 2 key targets (*PDK1* and *OARD1*) to construct prognostic risk prediction models. Through the functional enrichment analysis, single-cell sequencing, and the related assessments of the immune microenvironment, we explored the possible molecular mechanism. At the single-cell level, we explored the key cell (B cell). These results provide a basis and reference for the clinical diagnosis and treatment of TGCT.

As a crucial signaling transducer in the AKT signaling pathway, *PDK1* belongs to the serine/threonine kinase family. It is a key regulator of intracellular signaling and development. Akt phosphorylation caused by *PDK1* can mediate cell growth and proliferation or activate downstream mTOR signaling (34). *PDK1* could promote tumor proliferation and invasion through the PI3K/AKT/mTOR pathway in various types of cancer (35). It could also promote radioresistance in hepatocellular carcinoma cells (36). In this study, *PDK1*was lowly expressed in the HRG. These findings suggested that reduced PDK1 expression may contribute to tumor progression and immune evasion of neoplastic cells (37).

*OARD1* functions as an adenosine 5′-diphosphate (ADP)–ribosylhydrolase that removes protein ADP ribosylation. ADP-ribosylation abnormality was positively correlated with the tumor genomic instability. *OARD1* deficiency may likely cause defects in DNA repair and facilitate tumor progression. In this study, *OARD1* was first identified to be downregulated, which was associated with poor prognosis of seminoma. The low expression of OARD may impact the DNA damage response and repair.

In this study, both PDK1 and OARD1 exhibited negative coefficients in the risk model and lower expression in tumor tissues. This contradicts the classical pro-tumorigenic role of PDK1 typically observed in the PI3K/AKT pathway in other solid tumors. The specific “low expression - high risk” association in seminoma can be elucidated from three perspectives. First, seminomas originate from germ cells and reside in a highly tissue-specific tumor microenvironment, suggesting that PDK1 may possess non-classical functions distinct from those in somatic solid tumors (38). Second, atypical or reverse regulatory modes within the PI3K/AKT pathway may exist in seminomas, where low PDK1 expression could trigger the compensatory activation of downstream pro-tumorigenic bypass pathways through the relief of negative feedback loops (39). Finally, our supplementary clinicopathological analyses revealed that PDK1 and OARD1 expressions are independent of classical TNM staging or metastasis. These collective findings strongly imply that PDK1 and OARD1 should be defined as protective or differentiation-related prognostic markers representing the intrinsic molecular state of the seminoma, rather than direct classical oncogenic drivers.

The results showed the AUC values for 1, 2, and 3 years in the training set were 0.62, 0.66, and 0.60, respectively. Compared with the study of its kind, this model showed better predictive performance (40). This model could be used as an independent prognostic evaluation index for patients. Despite the other AUC values being higher than this study, the study revealed a new perspective about the integration of chemical-gene interaction in prognosis.

GSEA revealed significant enrichment of hormone-related signaling pathways in the HRG, particularly steroid hormone biosynthesis and related metabolic pathways. Previous studies have demonstrated that RNA regulatory networks, particularly lncRNA-miRNA-mRNA interactions, play essential roles in steroid hormone signaling and cancer progression (41). Furthermore, histidine, a semi-essential amino acid, is widely involved in various metabolic pathways and plays crucial roles in numerous physiological processes. Histidine metabolism is closely associated with the occurrence and development of numerous clinical diseases. Histidine is decarboxylated by histidine decarboxylase (HDC) to produce histamine. A main metabolic pathway of histamine leads to the formation of imidazoleacetic acid. Histidine can eventually be metabolized to form glutamate, which is then converted to &alpha; -ketoglutarate by deamination via glutamate dehydrogenase. In addition, GSVA analysis suggested that the histidine metabolism pathway was activated in the HRG. Histamine activates H1 receptors on the macrophages to promote the polarization of macrophages toward the M2 type. And this situation may reduce the antitumor immune response. These findings suggest that increased histidine metabolism may contribute to immune evasion through M2 macrophage-mediated immunosuppression, potentially explaining the poor prognosis observed in the HRG and aligning with our previous immune infiltration analysis.

Immune infiltration analysis showed that the HRG was highly correlated with macrophage infiltration. Macrophages and activated mast cells were remarkably higher in HRG than in LRG, while the HRG had fewer plasma cells, activated T cells, CD4 memory, and γ δ T cells than the LRG. These observations may be associated with DNA methylation for some specific genes.

Analysis of regulatory networks may provide important hints that lncRNA-miRNA-mRNA may be involved in the progression of seminoma through *PDK1* and *OARD1*. Recently, it was shown that the miRNA-mRNA interactions in GC impact the AKT in the P/A/M pathway to promote the development of gastric cancer. We hypothesize that a similar mechanism contributes to seminoma.

The 3D molecular docking and MD simulation studies have introduced important complementary hypotheses regarding the potential interactions between PDK1, OARD1, and ATBC. The structural matching, stable binding energies (-6.5 and -5.5 kcal/mol), and robust thermodynamic stability observed over 100 ns MD simulations suggest a high theoretical probability of interaction. Specifically, the formation of stable hydrogen bond networks indicates that ATBC might influence the allosteric or catalytic sites of these proteins. It is hypothesized that such binding might interfere with the regulation of downstream PI3K/AKT signaling or ADP-ribosylation functions (42). However, it must be emphasized that molecular docking and MD simulations only provide predictions of potential intermolecular binding. They cannot definitively prove real intracellular binding, target regulation, or actual toxicological effects (43). Therefore, the current computational results should be treated strictly as a supplementary hypothesis rather than mechanistic validation. Future comprehensive functional experiments, including the detection of PDK1/OARD1 expression, AKT phosphorylation, and cellular phenotypic assays in seminoma cells after ATBC treatment, are indispensable to substantiate these mechanistic predictions.

scRNA-seq has discovered 5 cell type identifications, 1 of which was identified as the key regulator. The B cells may contribute to antitumor immunity by directly damaging cancer cells, generating tumor-specific antibodies, and promoting tumor antigen uptake by DCs. In addition, B cells may also contribute to T cell activation via the secretion of cytokines such as interferon (IFN)‐γ, IL‐10, tumor necrosis factor (TNF), and IL‐6 (44, 45). Cell-cell communication analysis revealed that the B cells in the HRG may regulate the expression of MMP9 and CTSK in macrophages to affect the invasive capability of cells and, subsequently, tumor progression. At the same time, the interaction may form Lymphoid follicles in tumor microenvironments. It may be involved in cancer immune escape (16). The time analysis showed that the expression of *PDK1* and *OARD1* dynamically changes during cellular differentiation. It is suggested that *PDK1* and *OARD1* may regulate B-cell polarization to influence tumor progression. This analysis also provides new ideas and targets for the clinical treatment.

In this study, ATBC target genes were first associated with seminoma. We found 2 prognostic genes (*PDK1* and *OARD1*) and revealed the possible mechanism of tumor progression. Further analysis at the single-cell level identified the B cell as a critical regulator cell. However, the present study presents some limitations. First, to ensure sufficient statistical power for the LASSO algorithm (avoiding overfitting due to small sample size), the prognostic model was constructed using the entire TCGA-TGCT cohort, which inevitably included non-seminoma subtypes. Although the target genes were rigorously screened from pure seminoma datasets and mixed germ cell tumors often contain seminoma components, the biological heterogeneity among TGCT subtypes might introduce certain confounding effects. Nevertheless, the successful performance of the model suggests that these seminoma-derived markers possess broader prognostic applicability across the TGCT spectrum. Furthermore, although a certain discriminatory potential was shown by the model in the present study cohort, further validation is required in larger samples, independent cohorts and prospective studies before clinical application can be considered. Relatively low AUC values (0.62, 0.66, 0.60 for 1-, 2-, and 3-year survival) were obtained in the training cohort, which were close to random prediction. Thus, concerns about the robustness of LASSO variable selection were raised. Although better performance was observed in the validation cohort (1-year AUC = 0.79), further validation in larger and independent populations was required to improve model stability. *In vivo* animal model experiments are needed to further confirm the function of *PDK1* and *OARD1* in seminoma. A key challenge for future research is to define the mechanism by which *PDK1* and *OARD1* can regulate tumor cells in seminoma to provide solid evidence for clinical treatment. This study used multiple bioinformatics research methods to reveal that *PDK1* and *OARD1* are prognostic genes for testicular seminoma, which are of great significance for developing strategies to mitigate the adverse effects of ATBC on the male reproductive system. This study provides new methods and insights for understanding the impact of environmental pollutants on the occurrence and progression of malignant tumors, as well as for formulating public health strategies.

## Data Availability

The datasets presented in this study can be found in online repositories. The names of the repository/repositories and accession number(s) can be found in the article/**Supplementary Material**.

## Supplementary material

Supplementary Figure 1 Differential expression analysis of PDK1 and OARD1 across clinicopathological characteristics.

Supplementary Figure 2 Pathway enrichment analysis between the high- and low-risk groups.

Supplementary Figure 3 Molecular regulatory network analysis.

Supplementary Figure 4 Quality control analysis of single-cell sequencing data.

Supplementary Figure 5 Dimensionality reduction and annotation of single-cell data.

Supplementary Figure 6 Cell-cell communication analysis in 5 cell types.

Supplementary Table 1 List of target genes for ATBC.

Supplementary Table 2 GO/KEGG enrichment results.

Supplementary Table 3 PH test results.

Supplementary Table 4 GSEA enrichment results.

Supplementary Table 5 GSVA enrichment results.

Supplementary Table 6 Correlation analysis results of differential immune infiltration cells.

Supplementary Table 7 Differential immune infiltration cell correlation between genes PDK1 and OARD1.

Supplementary Table 8 Correlation analysis between genes PDK1 and OARD1 and miRNA.

Supplementary Table 9 ProTox-3.0 results.

Supplementary Table 10 ADMET lab 3.0 results.

Supplementary Table 11 ChemBlink results.

Supplementary Table 12 Enrichment pathways of different types of cells.
