# Supplementary figures and images for: Effective analysis of testicular seminoma toxicity and mechanisms of acetyl tributyl citrate using network toxicology, bulk RNA sequencing data, single-cell RNA sequencing data, and clinical data
Source: Front Immunol. 2026 Jul 13;17:1752528. doi: 10.3389/fimmu.2026.1752528 (PMC13402383; doi:10.3389/fimmu.2026.1752528)

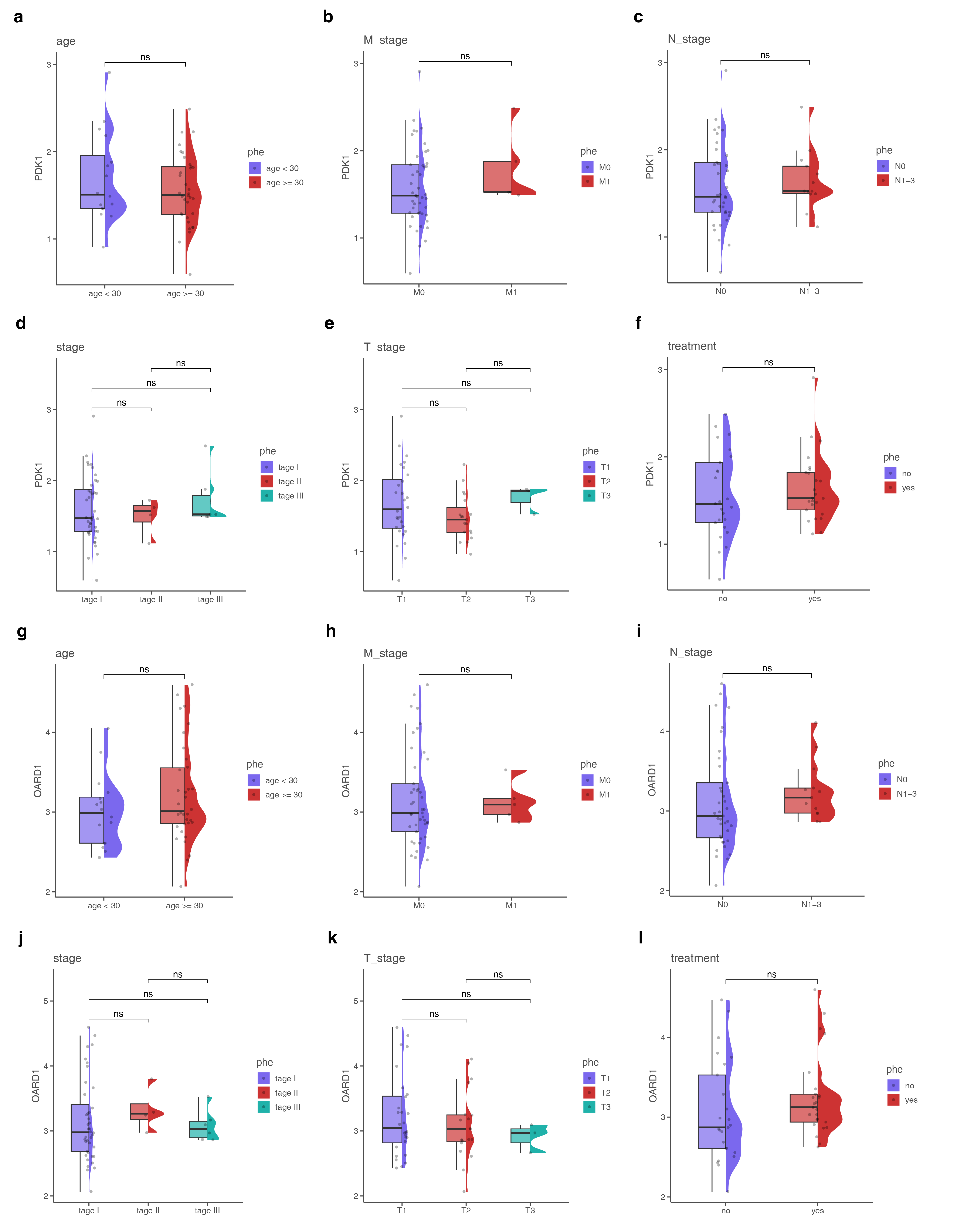

Supplement: Supplementary Figure 1 — Differential expression analysis of PDK1 and OARD1 across clinicopathological characteristics. [file Image1.tif]

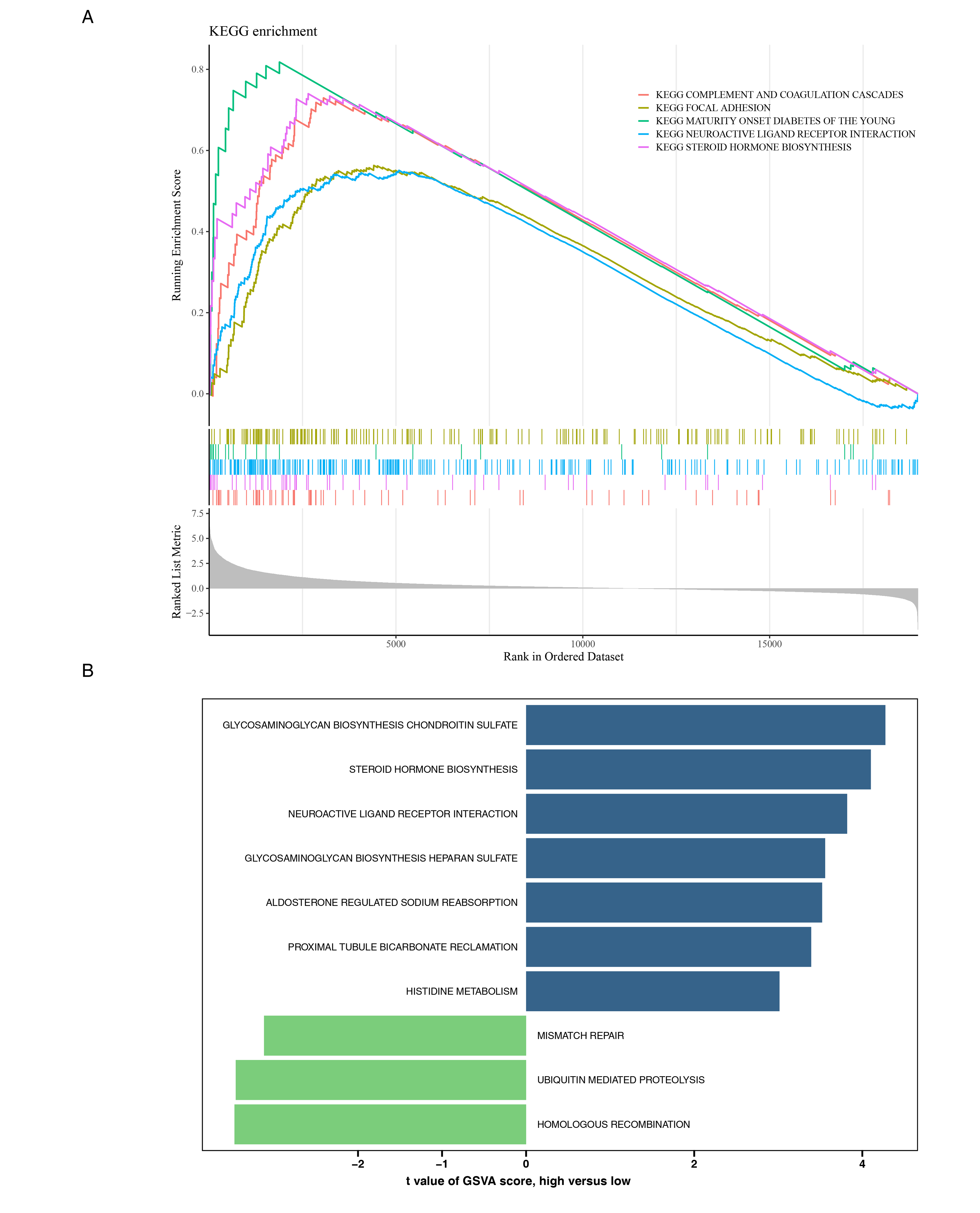

Supplement: Supplementary Figure 2 — Pathway enrichment analysis between the high- and low-risk groups. [file Image2.tif]

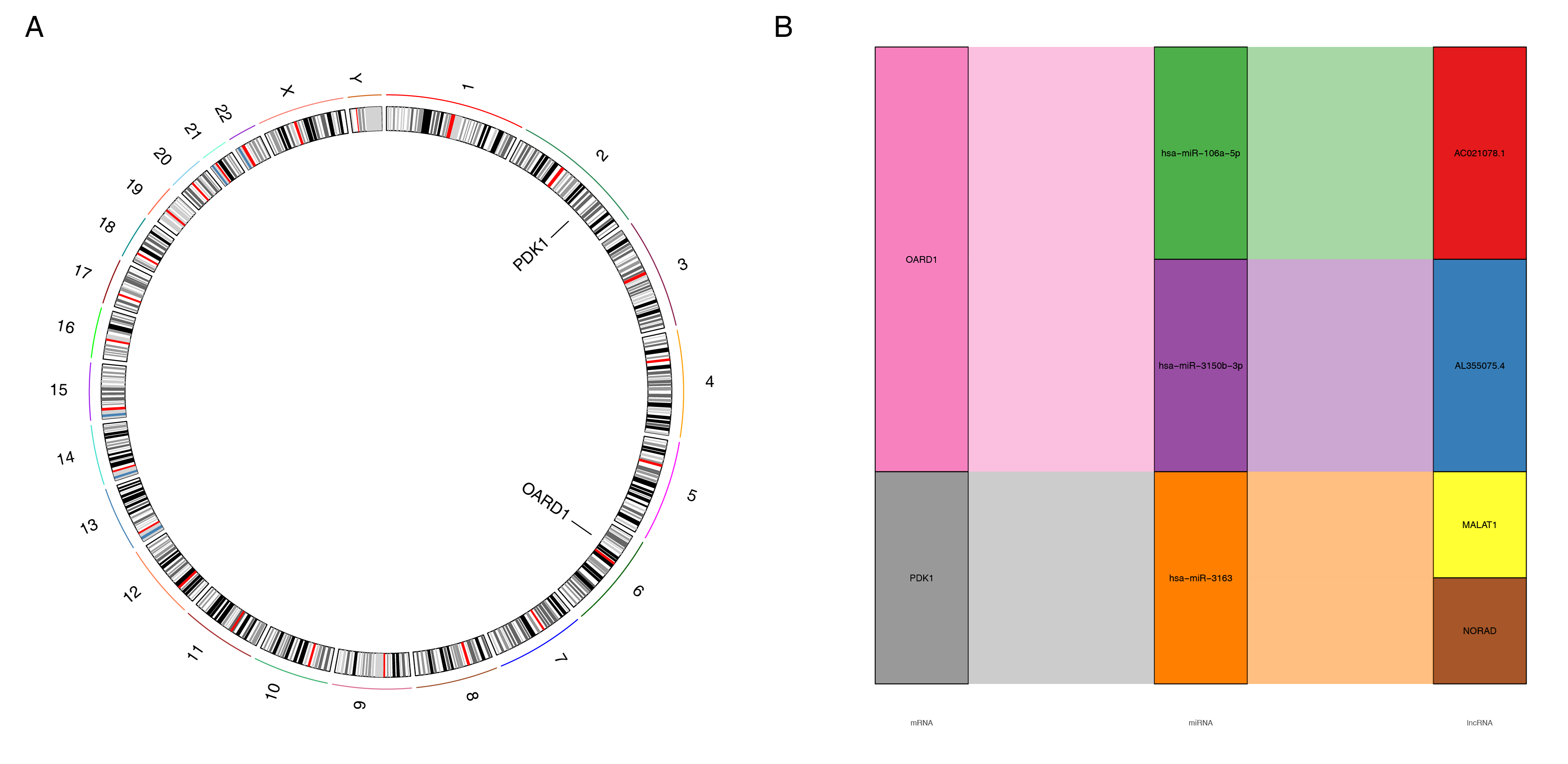

Supplement: Supplementary Figure 3 — Molecular regulatory network analysis. [file Image3.tif]

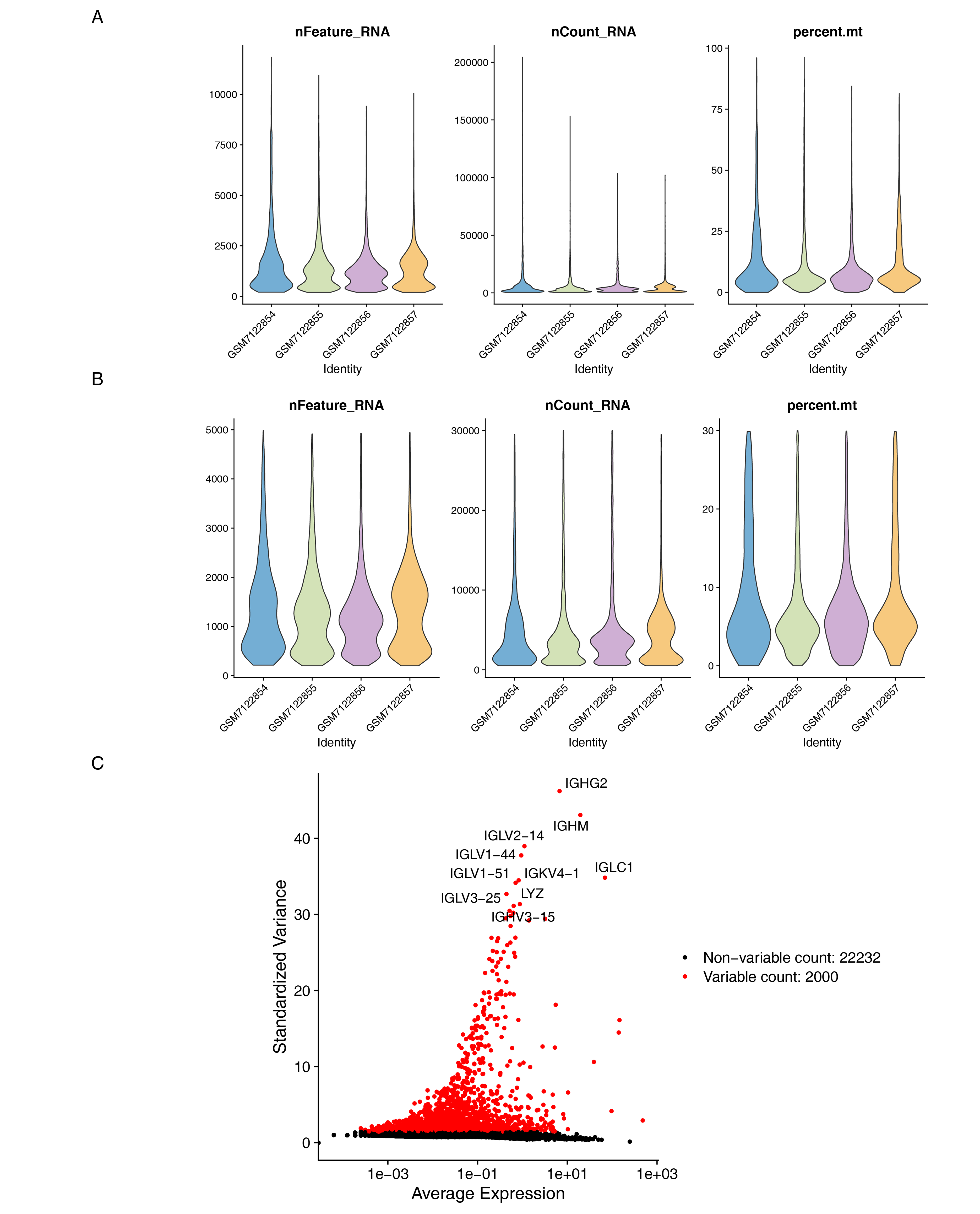

Supplement: Supplementary Figure 4 — Quality control analysis of single-cell sequencing data. [file Image4.tif]

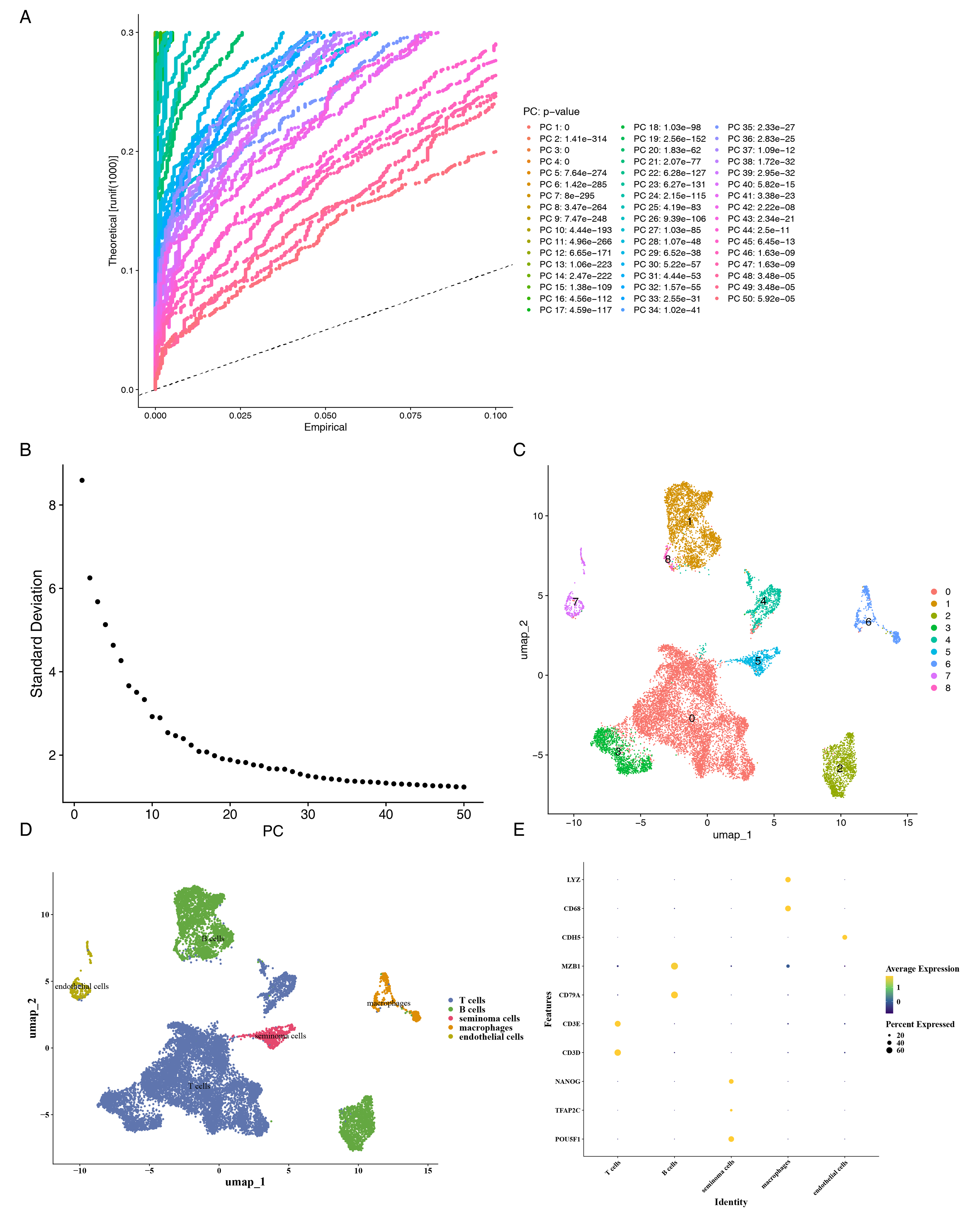

Supplement: Supplementary Figure 5 — Dimensionality reduction and annotation of single-cell data. [file Image5.tif]

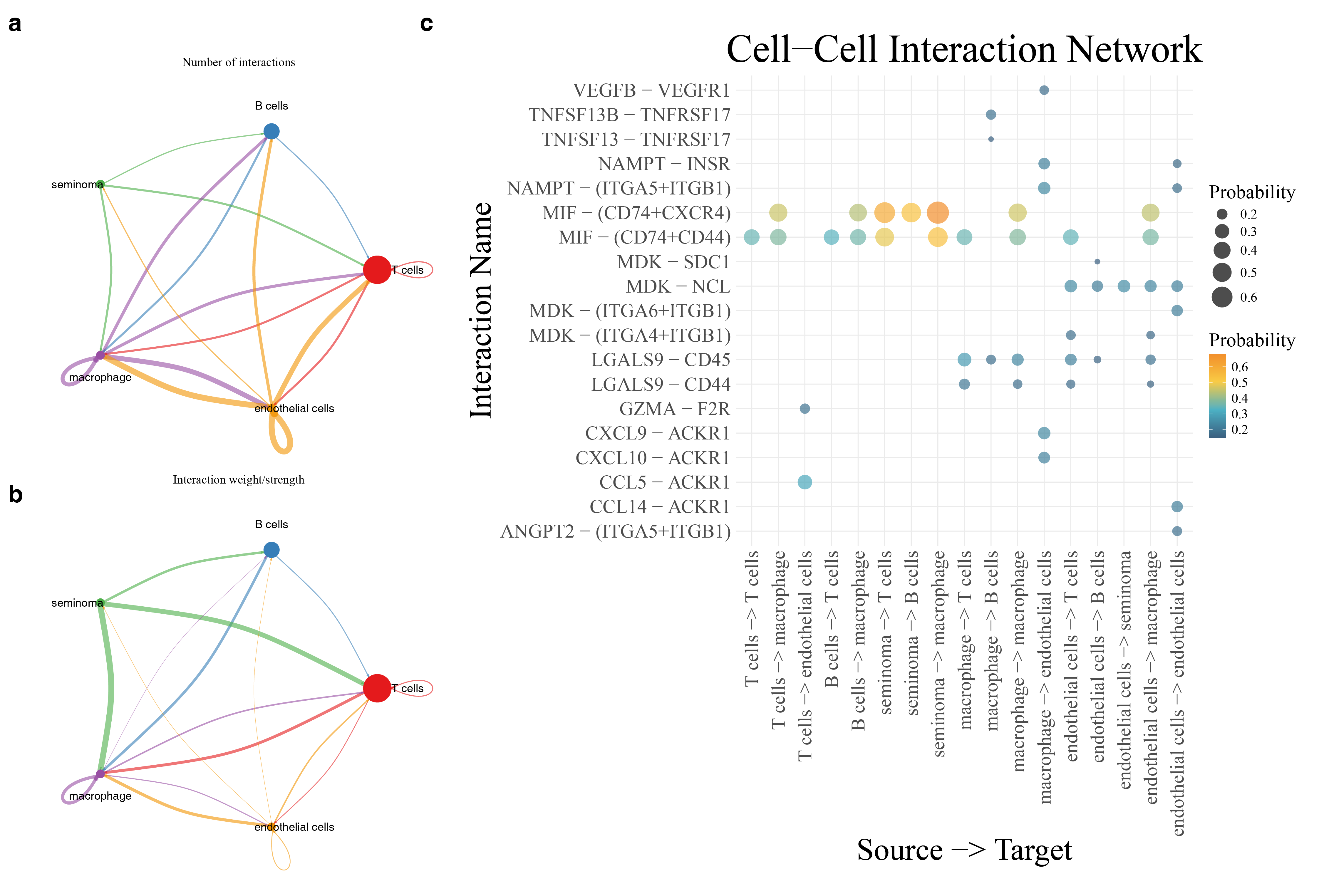

Supplement: Supplementary Figure 6 — Cell-cell communication analysis in 5 cell types. [file Image6.tif]
